# Surgical Treatment of Irreducible AcuteTraumatic Atlantoaxial Rotatory Subluxation in an Adult

**DOI:** 10.7759/cureus.31678

**Published:** 2022-11-19

**Authors:** Mutasim H Alhasani, Moutasem M Obeidat, Abdulaziz A ALMohammed, Abdullah M Alotaibi, Saad Surur

**Affiliations:** 1 Orthopedic Surgery, King Saud Medical City, Riyadh, SAU

**Keywords:** trauma, subluxation, rotatory, cervical spine, atlantoaxial rotatory subluxation, atlantoaxial dislocations, atlantoaxial

## Abstract

Traumatic atlantoaxial rotatory subluxation (AARS) is a condition that is extremely rare in adults when compared to the pediatric population. The most common symptoms of this condition are torticollis and post-traumatic neck pain. Our patient is a 41-year-old male who presented to the emergency room within hours of the injury. He came by himself with his relative as a case of road traffic accident. He was the first passenger and had been restrained during the car accident, with no ejection or rollover. He presented with stiffness/pain and reduced range of motion in the neck. Computed tomography (CT) of the cervical spine showed rotatory subluxation of C1 over the C2 with a locked facet. Within 24 hours of the RTA and patient admission, we attempted cervical traction. The reduction was not successful. So, we decided to reduce AARS through a surgical approach. The patient was taken to the operating room for open reduction and fixation using the Harms technique for C1-C2 fusion. The patient recovered from the surgery uneventfully, without any complications, recovered cervical mobility, and improved torticollis. Surgical management through open reduction and internal fixation is recommended for AARD cases in which close reduction fails due to a locked facet.

## Introduction

Rotatory subluxation of the atlantoaxial joint is a trauma-related condition that involves inferior-atlanto and superior axial facet articulations [1,2]. This condition rarely affects adults.

Spine surgeons often struggle with the diagnosis of this condition [3]. Although the pathogenesis of this condition is unclear, Barcelos et al. argue that the inflammation and hyperemia that are typical of the pathogenic process cause an increase in the laxity of the anterior transverse ligament, which consequently leads to the subluxation of the joint [4]. Delays in treatment and diagnosis can lead to upper cervical cord injury-related mortality through respiratory arrest, neurological deficiency, or both [5,6].

C1-C2 rotatory subluxation is predominantly found in the pediatric population. It is usually caused by mild trauma or, in most cases, by a respiratory infection of the upper respiratory tract (Grisel syndrome) [7-9].

The condition is predominantly found in children because of the higher proportion of the size of their head relative to the body; the insufficient development of the neck muscles; the elasticity of the articular capsule and the large angle of rotation between C1 and C2; and the horizontal configuration of the facet joints between the atlas and axis [5,10,11].

In adults, this injury can be caused by high-energy trauma such as car accidents, falls, or sports accidents [7].

In this paper, we have described a rare case of a traumatic Type 1 C1-C2 AARS injury that occurred in a 41-year-old male who was involved in a road traffic accident (RTA). Following the failure of the initial conservative treatment given to the patient after one week, surgical treatment was given, involving open reduction/internal fixation.

## Case presentation

A 41-year-old male was presented to the ER as a case of RTA. He was the first passenger and had been restrained during the car accident, with no ejection or rollover. He presented with stiffness/pain and reduced range of motion in the neck.

**Figure 1:**
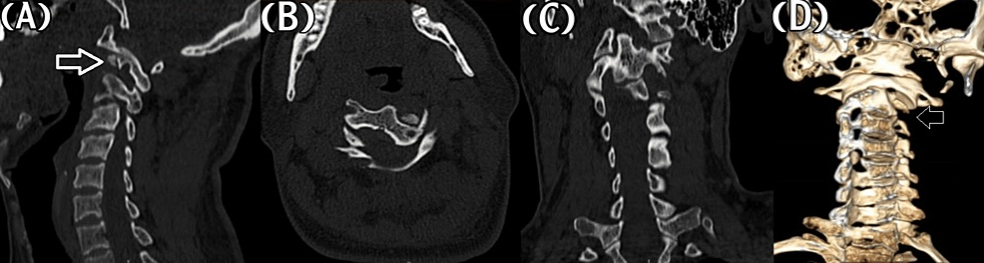
Preoperative CT of the cervical spine Sagittal (a), axial (b), and coronal CT (c), and CT with 3D (d) bone window. The CT images of the cervical spine demonstrate the left atlantoaxial rotatory subluxation (arrows), where the atlas rotated on the odontoid.

On examination, it was found that he had posterior neck tenderness and the torticollis Cock-Robin posture. Neurological examination revealed that the nervous system was intact. Computed tomography (CT) of the cervical spine with three-dimensional reformations showed an asymmetrical space between the odontoid process and lateral masses, which was widening on the left, with a displaced fragmented fracture of the left lateral portion of C2. It also indicated a possible partial extension of the C2 involving the left-sided foramen, which was suggestive of rotatory subluxation of C2 with a locked left C1 facet (Figure 1).

Neck CT angiography did not show any signs of vertebral artery injury. The cervical MR confirmed AARS with malalignment and left rotatory subluxation of C1. However, the transverse and alar ligaments were intact (Fielding type 1).

Within 24 hours of the RTA and patient admission, we attempted cervical traction. We applied skull traction using Gardner tongs and then increased traction weight up to 15 lb, along with appropriate administration of muscle relaxants, sedatives, and painkillers. However, the reduction was not successful. So, we decided to reduce the AARS through a surgical approach.

The patient was taken to the OR for open reduction and fixation using the Harms technique for C1-C2 fusion [12]. The surgical procedure was performed under general anesthesia, with the patient in a prone position. Gardner skull traction was applied, following which the standard posterior midline cervical spine approach was used to expose the upper cervical spine. After exposing the posterior arch of C1, a Penfield 4 was inserted under the C1 lateral mass at the left side and used as a joystick to gently manipulate and reduce the locked facet. Successful reduction was achieved with an audible pop sound, following which C1 lateral mass screws were inserted along with C2 pedicle screws. C3 lateral mass screws were also inserted as there was a destruction of the capsule and widening in the C2-C3 joint. The C1/C2 screws were fixed using rods.

The patient recovered from the surgery uneventfully and without any complications. Postoperative cervical spine X-ray confirmed the successful reduction of C1/C2 and good positioning of the screws.

**Figure 2:**
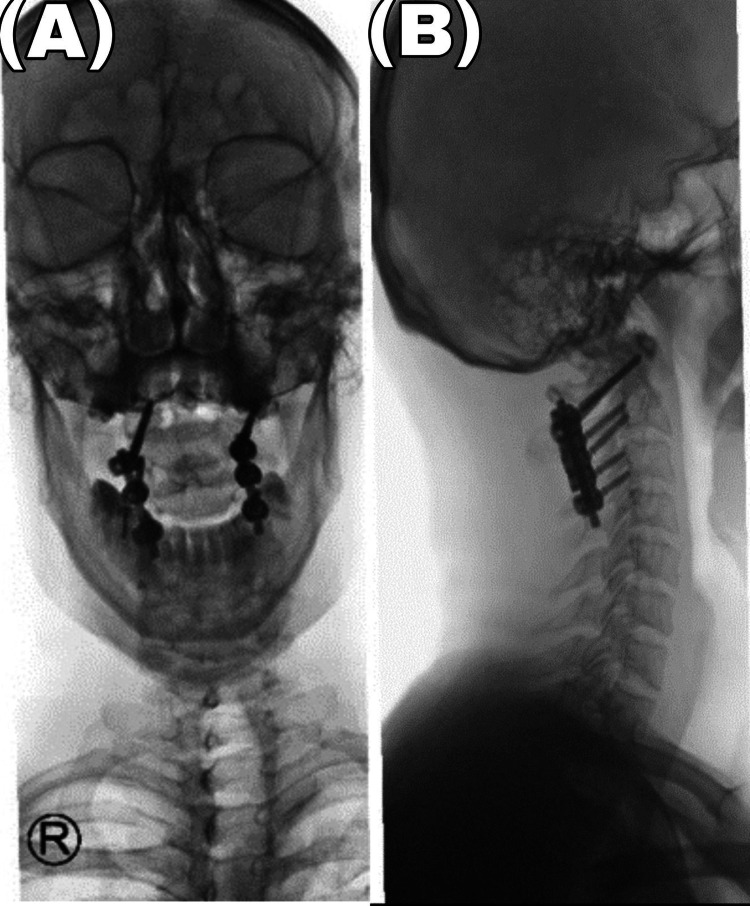
Lateral atlantoaxial spine radiograph during follow-up (a) Open mouth, (b) Showing the satisfactory alignment of C1 and C2 and good positioning of the screw

The three and six-month postoperative cervical films showed satisfactory C1-C2 alignment without any instability. The patient was also fully neurologically intact (Figure 2).

**Figure 3:**
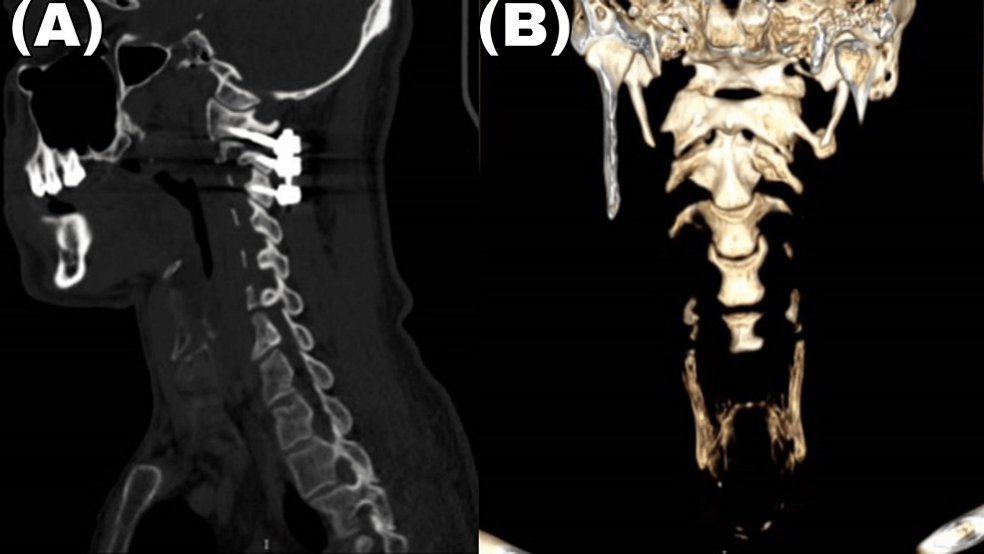
Postoperative CT of the cervical spine Sagittal (a) and CT with 3D (b). Bone window CT of the cervical spine after 10 months of surgery demonstrates a reduction of the locked facet and good alignment of C1 and C2.

X-ray and CT of the cervical spine taken during the 10-month follow-up showed maintained reduction, which meant that the clinical outcome was excellent. The patient had recovered cervical mobility and improved torticollis. He complained of only slight neck pain when engaging in intense exercise (Figure 3).

## Discussion

Rotatory subluxation of the atlantoaxial joint is a rare condition in adults, involving the articulation of the inferior-atlanto and superior axial facets [1,2].

Early detection and treatment of this condition are crucial for ensuring optimal neurological results. Although CT is still the gold standard for recording these injuries, patients with suspected AARS should also have an MRI taken, to get a more thorough assessment of the accompanying soft tissue damage (e.g., ligamentous injuries, and/or extent of spinal cord compression) [13,14].

The syndrome can also develop spontaneously or in conjunction with other disorders. Typically, either an infection or a stressful incident causes the condition. Ankylosing spondylitis-related rotatory abnormalities have also been reported in a few isolated case reports [15]. Metastatic cancer and eosinophilic granuloma can also occur following C1-3 laminectomy and suboccipital craniotomy [16-18].

The pathophysiology of AARF is not well-defined. About 50% of axial neck rotation is predominantly enabled by the atlantoaxial joint, and most of the biomechanical ligamentous stability is provided by the transverse and alar ligaments. The facet joint capsule and transverse ligament prevent the anterior translation of C1 on C2. The lateral aspect of the foramen magnum is connected bilaterally to the posterolateral apex of the odontoid by the alar ligaments, mainly limiting the anterior shift of the atlas on the odontoid and excessive rotation [8,15].

Children are more likely to suffer from AARF than adults because of their larger head-to-body ratio, underdeveloped neck muscles, the elasticity of the articular capsule, the great angle of rotation between C1 and C2, and the horizontal arrangement of the facet joints between the atlas and the axis [2,10,11].

Evidence from cadaveric dissection and magnetic resonance imaging studies suggests that rotatory subluxation can occur due to the disruption of the facet capsule followed by alar ligament disruption. The lateral mass of the atlas rotating posteriorly locks behind the ipsilateral lateral mass of the axis, causing severe forms of atlantoaxial rotatory instability [8,19].

Four types of AARF were described by Fielding and Hawkins. This categorization is widely accepted and corresponds with a higher risk of spinal instability and possible neurological damage. In type I AARF, the facet joints move without an increase in the alignment of the atlantodens interval. The only situation in which this can occur without rupturing ligaments is when the dens serves as a pivot and the rotation is within the typical range of normal atlantoaxial rotation. In type II AARF, the transverse ligament might tear, increasing the atlantodens gap by 3-5 mm. If the atlas is displaced bilaterally with a displacement greater than 5 mm, it would be type III and if the spinal canal does narrow, it would be type IV [17].

The most frequent symptom of AARF is cervical pain accompanied by the typical position of torticollis with lateral neck flexion and contralateral rotation position known as Cock-Robin [2,17].

There are different lines of treatment for AARF, including conservative and surgical management. The main goal of treatment is to relieve pain, restore spinal stability, and prevent the development of neurological deficits. The decision to adopt a surgical approach is taken based on the stability of the joint, its relocation, and the involvement of the transverse alar ligaments [20,21]. For cases of AARD with spinal instability, neurological involvement, or the inability to achieve or sustain reduction through conservative methods, a surgical approach is recommended [5,17]. A collar brace, cervical traction, or manipulation may help in reducing the dislocation if the damage was minor [5].

Definitive management of traumatic unilateral AARS would differ from case to case, as these injuries have special biomechanics and may often need a specific strategy for treatment [1].

In our case, a detailed radiological evaluation indicated that the lesion was consistent with type I AARD. Since the patient had no neurological deficit, we attempted cervical traction, which ended up in failure. We then attempted a surgical approach to reduce deformity, prevent the development of neurological deficits, relieve pain, and restore the range of motion in the neck.

## Conclusions

Surgical management through open reduction and internal fixation is recommended for AARD cases in which closed reduction fails due to a locked facet. It is also recommended for patients with type II AARD and more severe kinds of AARD as the transverse ligament damage can cause C1-2 instability.

In this paper, we have described a rare instance of type I post-traumatic AARD in an adult patient who was successfully treated through open reduction and C1-2 transpedicular screw fixation with no postoperative sequelae.
